# Identification of Potato *StPIN* Gene Family and Regulation of Root Development by *StPIN4*

**DOI:** 10.3390/ijms252111517

**Published:** 2024-10-26

**Authors:** Qian Zhang, Qing Liu, Jiangwei Yang, Ning Zhang, Huaijun Si

**Affiliations:** 1 State Key Laboratory of Aridland Crop Science, Gansu Agricultural University, Lanzhou 730070, China; 18409391944@163.com (Q.Z.); ningzh@gsau.edu.cn (N.Z.); 2 College of Life Science and Technology, Gansu Agricultural University, Lanzhou 730070, China; lq__677@163.com

**Keywords:** PIN-FORMED, potato, bioinformatics analysis, StPIN4, root architecture

## Abstract

The growth hormone export PIN-FORMED (PIN) is an important carrier for regulating the polar transport of plant growth hormones and plays an important role in plant growth and development. However, little is known about the characteristics and functions of PINs in potatoes. In this study, 10 *PIN* members were identified from potatoes and named *StPIN1*, *StPIN2*, *StPIN3*, *StPIN4*, *StPIN5*, *StPIN6*, *StPIN7*, *StPIN8*, *StPIN9*, and *StPIN10* according to their positions in the potato chromosome In addition, the expression of 10 *StPINs* was analyzed by qRT-PCR during potato root development. The results showed that the *StPIN4* gene plays an important regulatory role in potato root development, and its tissue expression varied greatly. Several *cis*-regulatory elements related to growth factors were also detected in the promoter region of the *StPIN* gene. The transgenic overexpressing *StPIN4* in potato showed suppressed growth in root length and lateral root number, and *StPIN4*-interfering plants showed the opposite. These results suggested that *StPIN4* plays a key role in the regulation of the potato root architecture.

## 1. Introduction

Growth hormones are widely found in plants and are involved in regulating various stages of plant growth [1]. The growth hormone is synthesized primarily in actively growing tissues, such as stem tips and root tips, and it acts in various tissues and organs primarily through polar transport [2]. Currently, growth hormone transporter proteins in *Arabidopsis* have been identified in the following three families: the growth hormone input carrier resistant/like *AUX1* (AUX/LAX), the output PIN-FORMED (PIN), and the multidrug-resistant/p-glycoprotein family (MDR/PGP) [3]. AUX/LAX is a universally recognized growth hormone export carrier encoding multi-transmembrane proteins, which is a subfamily of the amino acid transporter protein family. In *Arabidopsis*, the AUX/LAX family consists of four highly conserved genes, *AUX1*, *LAX1*, *LAX2*, and *LAX3*. Immunolocalization showed that the *AUX1* gene was expressed in root tip tissues and root epidermal cells, suggesting that the *AUX1* protein is expressed in the root tip. The *AUX1* protein is involved in the transport of growth hormone from the root tip to the root base, thus causing differences in growth hormone concentration between different sites. *LAX1*, *LAX2*, and *LAX3* were expressed in *Arabidopsis* root cell tissues and vascular tissues of aboveground parts and were involved in the growth hormone-ethylene interaction during seedling root tip development [4]. MDR/PGP is a subfamily of the ABC (ATP-binding cassette, ABC) transporter protein family, a subfamily of the ABC family of transporter proteins, which are also involved in the polar transport of growth factors. It is widely found in eukaryotic and prokaryotic organisms. It also participates in the polar transport of growth factors and plays a key role in a series of biochemical reactions, including intercellular signaling and regulation. The MDR1 protein is localized in tissues associated with apical transport in primary and lateral roots, and *PGP4* is expressed in root hair cells and functions as both an input and an output of growth-hormone transport [5]. The first member of the *PIN* family to be identified and associated with growth hormone transport, *AtPIN1*, was described in the model plant *Arabidopsis thaliana*. The significance and function of *AtPIN1* was discovered through the phenotypes generated by the mutation: the mutant plants were unable to develop normal floral organs and produce naked inflorescences, thus giving the family its name, PIN-FORMED (PIN) [6]. The PIN-FORMED (PIN) family of proteins are transmembrane transport proteins in plants [7]. In plant growth and development, the regulation of endogenous hormones cannot be ignored, especially the polar transport and asymmetric distribution of growth hormones. *PIN* forms a family of growth hormone efflux carrier genes that play a key role in the polar transport of growth hormones, which in turn regulate the growth and development of different plant tissues [8]. *PIN* genes are found only in the genomes of multicellular plants and play important roles in regulating the asymmetric distribution of growth factors, including embryogenesis, organogenesis, and tissue differentiation. All PIN proteins have a similar structure with amino- and carboxyl-terminal hydrophobic structural domains, and transmembrane structural domains separated by a central hydrophilic structural domain. The hydrophobic domain is structurally conserved. In contrast to the hydrophilic domain, the activity of PIN proteins is regulated at multiple levels, including transcription, protein stability, subcellular localization, and transporter activity [9].

PIN proteins are a class of transmembrane proteins consisting of two hydrophobic regions and a hydrophilic loop, which can be initially divided into two subgroups based on the length of the hydrophilic loop [10]. With the continuous development of sequencing technology, different numbers of PIN gene family members have been identified in a variety of plants so far. Among them, 8, 12, 29, 14, 17, 10, 15, 23, and 10 members were identified in *Arabidopsis thaliana* [11], rice [12], tobacco [13], corn [14], cotton [15], chili peppers [16], poplar [17], soybeans [18], and tomatoes [19], respectively. The members of the PIN gene family vary significantly between monocotyledonous and dicotyledonous plants, so the number of *PIN* genes appears to vary greatly between species. These differences better reflect the diversity and complexity of organisms in the process of evolution, so that different species are better adapted to changes in the environment. Moreover, several *PIN* genes have been shown to play important roles in plant rooting processes [20].

The adaptation of plants to their environment is also largely dependent on the roots, i.e., the underground parts [21]. In addition to the substances necessary for plant growth, the root system also plays an important role in plant development, as it anchors the plant in the soil so that it cannot only withstand various stresses but also better absorb nutrients [22]. Taking the model plant *Arabidopsis thaliana* as an example, the root system of the dicotyledonous plant *Arabidopsis thaliana* is divided into a primary root and an adventitious root, which develops from an embryonic root formed during embryogenesis, and after embryogenesis, the primary root can branch and form lateral roots [23]. That is, the primary root usually originates from the radicle and branches into several purposeful lateral roots; the adventitious root originates from the hypocotyl or root-hypocotyl junction [24]. Plant adventitious root development is mainly controlled by growth hormone activity and growth hormone response maximum [25]. PIN proteins play an important role in plant adventitious root formation by participating in the polar transport of growth hormones in plants. When the growth hormone content is higher than cytokinin, it mainly induces plant tissue dedifferentiation and root primordia formation, favoring root genesis. Specifically, growth hormones control root formation and development by regulating the balance of growth hormones and cytokinins [26]. PIN proteins display peculiar subcellular localization that determines the direction of growth hormone flow, and *PIN*-dependent plant growth hormone efflux plays a crucial role in morphogenetically asymmetric plant growth hormone distribution, which in turn controls plant growth and development. In the root tip, overexpression of *PIN1* results in the accumulation of growth hormone in root meristematic tissues, conversely, growth hormone content is reduced when *PIN1* expression is suppressed decrease [27]. Some researchers have pointed out that PIN-mediated polar growth hormone transport promotes root bending during obstacle avoidance, i.e., an asymmetric growth hormone response occurs in the roots during obstacle avoidance, polar growth hormone transport mediates the accumulation of growth hormones locally, and the root system bends away from the obstacle and finds routes to grow into deeper soils during a process known as obstacle avoidance, and that this tendency helps plants to efficiently immobilize themselves and find nutrients and water to survive in harsh conditions [28].

The potato (*Solanum tuberosum* L.) is an important dietary crop grown in many countries and regions of the world and is rich in a variety of vitamins and minerals that are beneficial to human health [29]. The potato is a typical shallow-rooted plant, and the growth of the root system has an irreplaceable role in potato yield and quality. Many plant *PIN* family genes have been identified, and it has been proved that some members of the *PIN* family play important roles in the formation of adventitious roots, but the specific functions of potato *PIN* family members in the formation of adventitious roots are still unclear. In this study, we screened candidate genes related to potato root development through genome-wide identification and bioinformatics analysis and transformed the potato to obtain transgenic lines. The transgenic lines were obtained by transforming the potato. The structural characteristics and potential functions of these genes were understood, and the observation and measurement of the root system of transgenic plants revealed the role of PIN-related genes in regulating the morphogenesis of the potato root system, which provided the theoretical basis and genetic resources for the further use of genetic engineering to reshape the root system of the potato to cultivate high-yielding and drought-tolerant potato varieties, and laid the foundation for the subsequent verification of the functions of potato *PIN* genes and the molecular mechanism of adventitious root formation.

## 2. Results

### 2.1. Identification and Analysis of Potato StPIN Family Genes

The Arabidopsis PINs sequences were downloaded from the Arabidopsis database (https://www.arabidopsis.org/index.jsp accessed on 4 October 2024) as reference sequences, after BLAST and Pfam structural domain analyses, 10 StPINs sequences were retrieved from the potato genome database, which was named StPIN1~StPIN10 (Table 1). Physicochemical properties of 10 identified members of the potato StPINs protein family analyzed using ExPASy (https://www.expasy.org/), the amino acid number ranged from 235 to 745, and the molecular weights of the original proteins ranged from 25,398. 31 to 81,967.28, with *StPIN1* having the smallest molecular weight, *StPIN7* having the largest molecular weight, and isoelectric point ranging from 6.57 to 9.22, using PSORT (http://psort.hgc.jp/) to predict the subcellular localization of the family and found that all 10 family members are located on the cytoplasmic membrane.

### 2.2. Chromosomal Localization and Gene Structure Analysis of Potato PIN Gene Family Members

**Figure 1:**
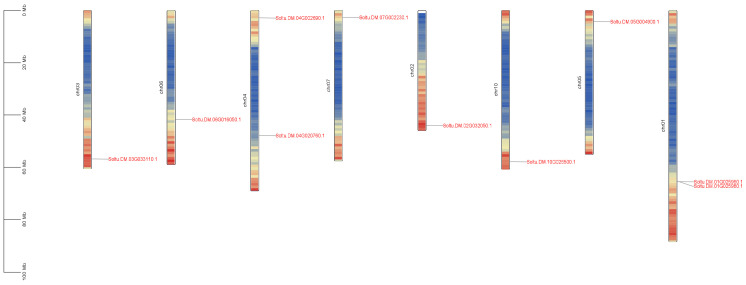
Chromosome location analysis of *StPIN* gene family. The different colors represent the density information of the genes, red represents higher gene density, and blue is the opposite.

**Figure 2:**
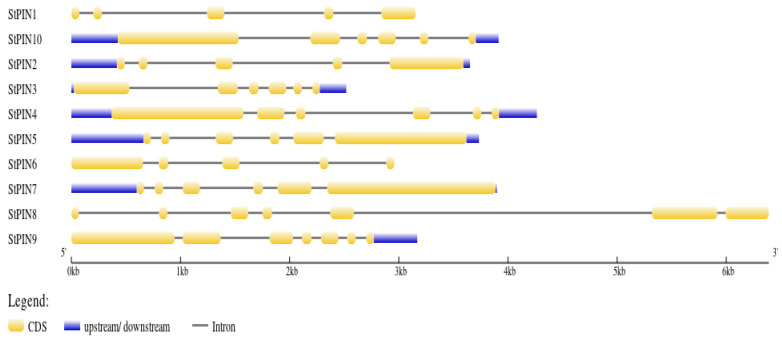
Gene structure characteristics of *StPINs*. The blue and yellow boxes represent UTR and CDS, and the black lines represent introns.

By sequence analysis, 10 *StPINs* genes were unevenly distributed on eight chromosomes (Chr1, Chr2, Chr3, Chr4, Chr5, Chr6, Chr7, and Chr10) of a potato (Figure 1). Among them, *StPIN* genes were found on chromosomes 1 and 4, and one member was distributed on each of the remaining six chromosomes. Gene structure analysis showed that among the 10 gene members, *StPIN3*, *StPIN4*, *StPIN5*, *StPIN7*, *StPIN8*, and *StPIN10* had five introns, *StPIN1*, *StPIN2*, and *StPIN6* had four introns, and *StPIN9* had six introns (Figure 2).

### 2.3. Potato StPINs Protein Family Motif and Phylogenetic Analysis

**Figure 3:**
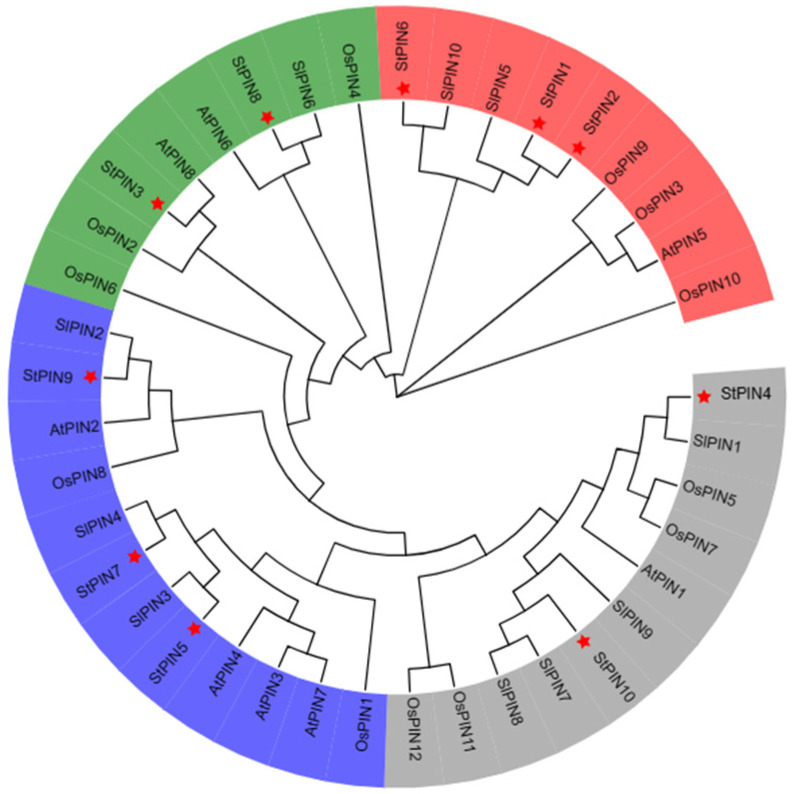
Unrooted phylogenetic tree of PIN proteins from *Solanum tuberosum*, *Solanum lycopersi cum*, *Arabidopsis thaliana*, and *Oryza sativa*. Each color represents one group. The red stars represent the highlighting of the *StPINs*.

**Figure 4:**
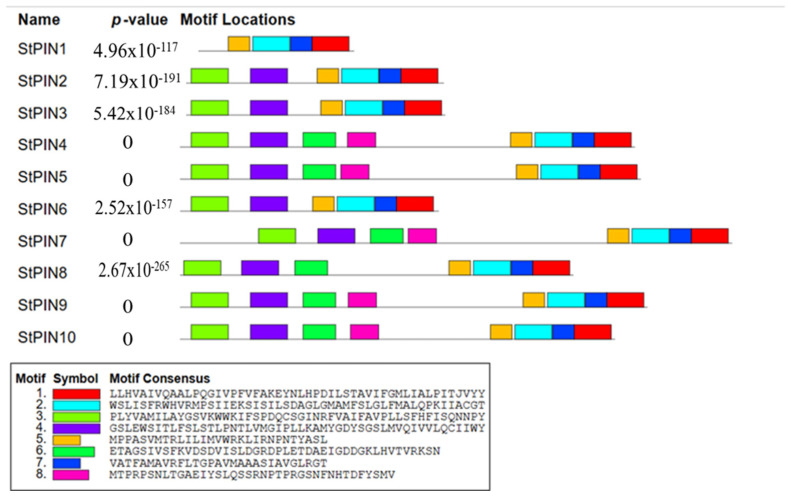
Conserved motifs of *PIN* genes in potatoes. MEME software (V5. 5. 4) facilitated the prediction of conserved motifs, with the motif quantity fixed at ten. One color box corresponds to one motif.

To investigate the evolutionary relationships of *PIN* gene families, phylogenetic analyses of the identified family members in potatoes, *Arabidopsis*, tomatoes, and rice were carried out using MEGA7 software (Figure 3). By comparing their affinities with those of other species, we found that the *StPINs* were most closely related to the tomato, e.g., *StPIN4* was highly homologous to *SlPIN1*, and *StPIN5* was highly homologous to *SlPIN3*. Next, we analyzed the conserved motifs among the 10 *StPIN* gene family members (Figure 4): *StPIN2*, *StPIN3*, and *StPIN6* all contained six conserved motifs; *StPIN4*, *StPIN5*, *StPIN7*, *StPIN9*, and *StPIN10* contained eight conserved motifs; *StPIN8* had seven conserved motifs; *StPIN1* and *StPIN8* had seven conserved motifs; *StPIN1* had the least number of conserved motifs, only 4. Visual analysis of the conserved motifs shows that the *StPINs* gene family members are relatively conserved among each other.

### 2.4. Analysis of Cis-Acting Elements of Potato PIN Gene Family Members

**Figure 5:**
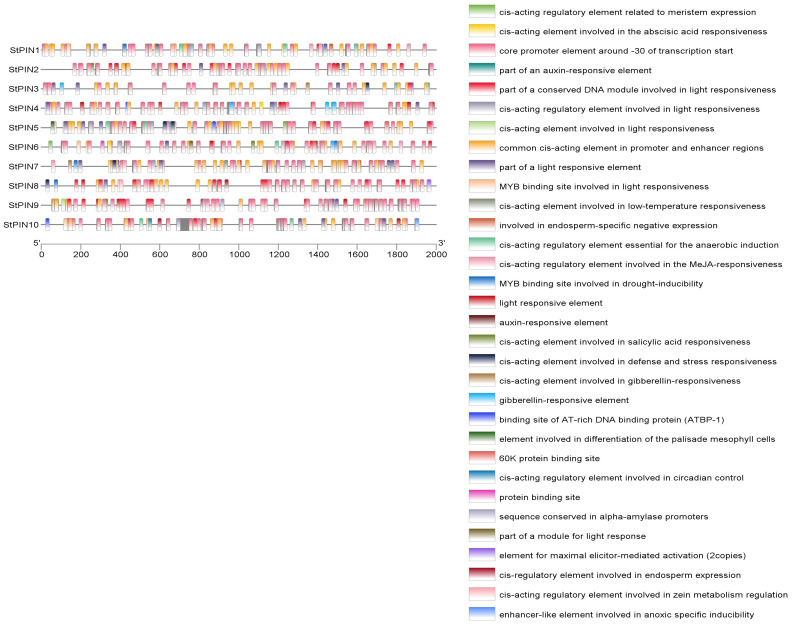
The *cis*-elements in the promoters of potato *PIN* genes. The PlantCare website was used to analyze and present the results with TBtools. The 2000 bp upstream of the *StPINs* was used to perform *cis*-elements analysis.

To study the response of the potato *PIN* gene family to hormones, we analyzed a 2000 bp sequence upstream of the *PIN* gene (Figure 5), which showed that all gene members contain the basic *cis*-acting elements CAAT-box and TATA-box, in addition to hormone-responsive elements: the genes *StPIN1*, *StPIN2*, and *StPIN6* contain the TGA-element, *StPIN1*, *StPIN2*, *StPIN4, StPIN6*, and *StPIN7* contain the methyl jasmonate (MeJA)-responsive element TGACG-motif, and *StPIN1*, *StPIN2*, *StPIN6*, *StPIN7*, and *StPIN9* contain the salicylic acid-responsive element TCA-element. *StPIN1*, *StPIN2*, *StPIN6*, *StPIN7,* and *StPIN9* contain the salicylic acid response element TCA-element, while the other genes contain the abscisic acid response element ABRE and the gibberellin response-related elements (TATC-box and P-box); in addition, they contain the light response element G-Box and the adversity defense response element. This suggests that the *StPINs* gene family is mainly hormone-regulated in potatoes, and is closely related to plant growth and development, which may be realized through the co-regulation of growth hormone and other hormones to regulate plant growth and development.

### 2.5. Expression of StPIN Gamily Genes During Potato Root Development

**Figure 6:**
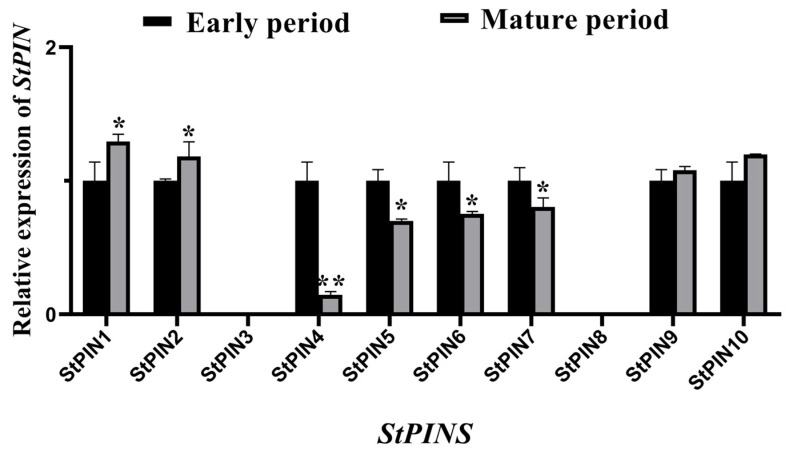
Expression pattern of *StPINs* in potato root development. Asterisks denote significant differences from control (* *p* < 0.05; ** *p* < 0.01).

The expression of 10 *StPINs* was analyzed by qRT-PCR at the early stage of potato root development and maturity, and the results are shown in Figure 6. *StPIN3* and *StPIN8* were not expressed during the development of the root system. Four genes were significantly down regulated in expression after root maturation compared to the early stages of development. Among them, *StPIN4* was down regulated most significantly.

### 2.6. Tissue Differential Expression Analysis of StPIN4 Gene

**Figure 7:**
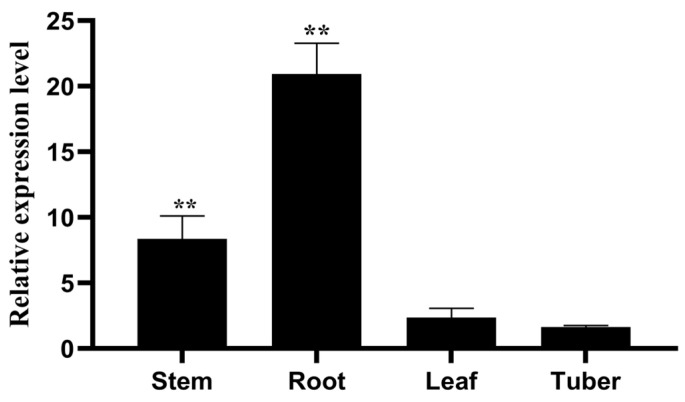
Tissue-specific expressions analysis of *StPIN4* gene in potato cultivar ‘Atlantic’. Asterisks denote significant differences from control (** *p* < 0.01). Method of statistical analysis of ANOVA.

To analyze the differences in the expression of *StPIN4*, different tissues were collected for tissue-specific expression analysis, and according to the results of qRT-PCR (Figure 7), the *StPIN4* gene was expressed in potato roots, stems, leaves, and tubers, but the highest expression was found in the roots, followed by the stems and leaves, and the lowest expression was found in the tubers. The expression in the roots was about 20 times higher than in tubers.

### 2.7. Acquisition and Assay of Transgenic Plants

**Figure 8:**
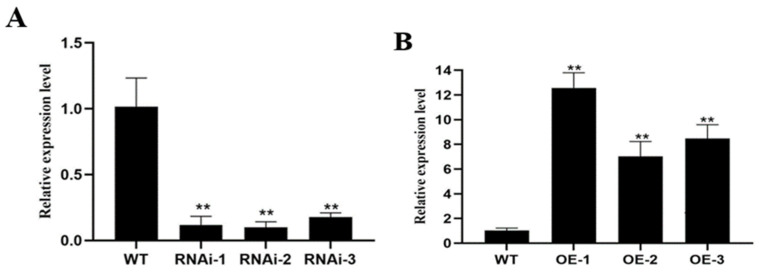
Expression analysis of *StPIN4* in WT and transgenic lines. *StEf1*α (elongation factor 1α) was used as an internal control. (**A**) Comparison of the expression levels of interfering expression lines and WT controls. (**B**) Comparison of the expression levels of overexpressed *StPIN4* lines and WT controls. Asterisks denote significant differences from control (** *p* < 0.01). Method of statistical analysis: Student’s *t*-test.

To further elucidate the regulation of potato root development by *StPIN4*, we performed a genetic transformation and obtained overexpression (OE) and interference expression (RNAi) lines of potato *StPIN4*. The qRT-PCR analysis was performed to further characterize the transgenic plants, and we identified the following six independent transgenic lines via analysis (Figure 8). The qRT-PCR analysis showed that *StPIN4* was significantly higher in overexpressing lines than in non-transgenic plants. However, the relative expression of *StPIN4* in the interference-expressing lines was significantly lower than that of non-transgenic plants. Based on the results of qRT-PCR analysis, we followed up the three existing overexpression lines (OE-1, OE-2, and OE-3) and three suppressor-expression lines (RNAi-1, RNAi-2, RNAi-3) for further analysis.

### 2.8. qRT-PCR Analysis and RNA Validation

The result of qRT-PCR melting curve analysis showed that there was a single smooth curve with a TM of the target gene around 80 °C (Figure S1). The extracted RNA was assayed by electrophoresis, and the ratio of 28S RNA to 18S RNA was about 2:1, and the bands of 28S RNA were brighter than those of 18S RNA, indicating that the extracted RNA was not degraded (Figure S2).

### 2.9. Phenotypic Assay of Transgenic Potato

**Figure 9:**
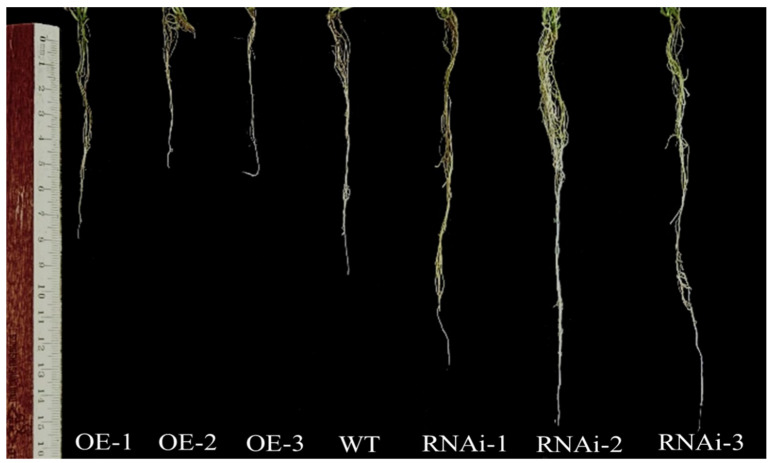
Phenotypes of the transgenic potatoes with *StPIN4* transformation. Root length: distance from the dotted line to the root tip.

**Figure 10:**
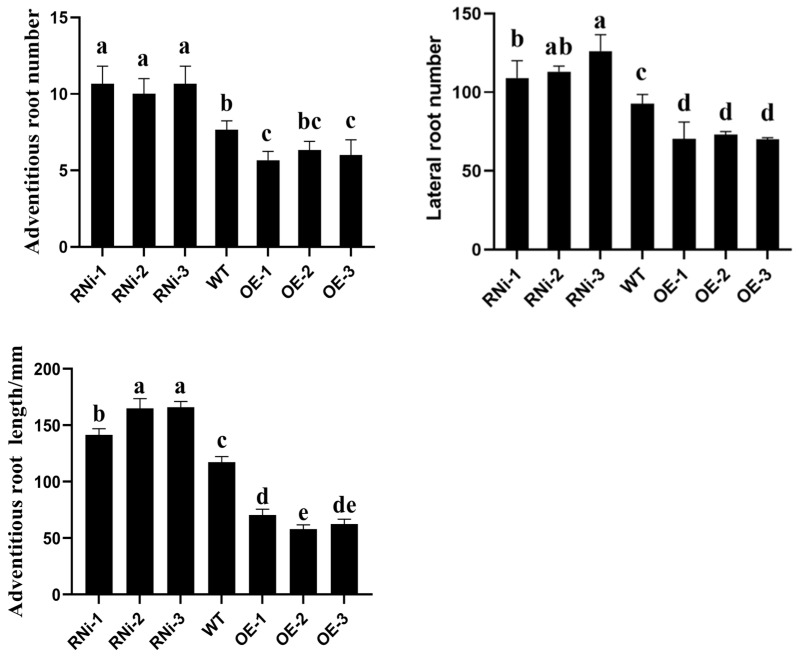
Phenotypic characteristics of transgenic potato plants and non-transgenic. The error bars represent the standard errors (*n* = 3). Different small letters mean significant differences (*p* < 0.05). Method of statistical analysis: ANOVA.

The transgenic plants from the culture for about 21 days were planted in 10 cm × 10 cm plastic pots and watered regularly, and the transgenic lines were observed and analyzed after 45 days. The results showed that there were no significant differences in the suppression of expression plants compared to non-transgenic plants under control conditions. In addition to that, statistically analyzed root morphology confirmed the significance of potato transgenic lines (Figure 9). Compared to the control non-transgenic strain, the overexpression lines showed significant inhibition of root growth and significantly shorter root lengths. In contrast, the number of primary roots of the interfering expression lines decreased significantly, the number of roots increased significantly, and the number of lateral roots also increased significantly (Figure 10).

## 3. Discussion

The plant root system is critical to plant growth. It has a wide range of functions, including nutrient and water acquisition, anchoring, and symbiosis with beneficial microbial communities in the soil to improve the efficiency of nutrient uptake [30]. Therefore, root architecture is an important agronomic trait directly related to crop yield. A well-developed root system can absorb water and nutrients more efficiently, thus supporting higher crop yields. In addition, the root system architecture is highly plastic, which largely determines the plant’s ability to adapt to changes in the external environment and enhances the overall health of the crop, thereby increasing its resilience [31]. Growth hormone is an important phytohormone that plays a decisive role in the plastic adaptation of plants to changes in the external environment, and *PIN* plays an irreplaceable role as a growth hormone transporter in the growth and development of plants. Numerous studies have shown that members of the *PIN* gene family regulate the formation of adventitious roots [15]. The number of *PIN* gene family members varies considerably among species. Among them, the number of *PINs* in tobacco was the highest, containing 29 members [13], and *Arabidopsis* had the least, containing 8 members [11]. In this study, 10 members of the *PIN* gene family were identified from potatoes, and these 10 members were comprehensively analyzed, and the results showed that except for *StPIN1*, *StPIN7*, and *StPIN9*, the other seven members are stable proteins, and most of the genes are composed of basic amino acids, among which, *StPIN1* has the lowest number of amino acids, containing only 233 amino acids, and *StPIN7* has the highest number of amino acids, containing 745 amino acids (Table 1). The functions of genes can be inferred based on the affinities of different species; therefore, the PIN protein families of three species, *Arabidopsis thaliana*, tomato, and rice, were selected as reference sequences, and the potato and tomato are more closely related to each other than to two species, *Arabidopsis thaliana,* and rice. Based on this, it was hypothesized that these proteins have similar functions during plant development. The gene structures of the potato *PIN* family members are similar, with each member containing motifs 1, 2, 5, and 7 (Figure 4). In addition, different groups have similar motifs, suggesting that these conserved motifs play an important role in specific functions. Among them, *StPIN9* and *StPIN10* genes are more similar in structure, physicochemical properties, and transmembrane structure, which suggests that they may have similar functions. The analysis of *cis*-acting elements revealed that the promoter region of this family contains hormone-responsive cis-acting elements, which mainly include growth hormones, gibberellin, and abscisic acid-responsive elements (Figure 5). It was hypothesized that the growth hormone-responsive cis-acting elements might be involved in the regulation of adventitious root formation with *PIN* family members [32].

Several members of the *PIN* gene family are involved in growth hormone transport between cells of plant tissues such as roots, stems, and cotton fibers [33]. In *Arabidopsis, AtPIN1*, *AtPIN2*, *AtPIN3*, *AtPIN4*, and *AtPIN7* are involved in intercellular growth hormone transport in the root system [9]. *AtPIN1* and *AtPIN3* are highly expressed in roots, determine the size of the primary root meristematic tissue, and determine the growth rate of the primary root [31]. *PIN10* and *PIN13* were expressed at higher levels in the roots of apples, suggesting that *PIN* content may play a major role in regulating root growth and development in apples [34]. In cotton, *PIN1*, *PIN2*, and *PIN3* were found to be involved in the development of the cotton root system and to play an important role in the formation of primary and lateral roots [15]. In this study, we analyzed the expression of 10 *StPINs* in potato roots at the early stage of development and maturity by qRT-PCR (Figure 6). The results showed that, except for *StPIN3* and *StPIN8*, which were not expressed, the other 8 *StPIN* genes were expressed in different degrees in the root system, and the expression of *StPIN1* and *StPIN2* were slightly higher in the mature stage than in the early stage of development, so these two genes may play a certain role in the mature stage of root development, while the expression of *StPIN4*, *StPIN5*, *StPIN6*, and *StPIN7* was significantly higher in the predevelopment stage than in the mature stage of root development, so we hypothesized that these four genes have an important role in the predevelopment stage of the potato root system. Among them, *StPIN4* was more significantly expressed in the early and mature stages of root development than the other seven genes, so we chose *StPIN4* as the target gene for the subsequent study. Furthermore, the expression of *StPINs* at different developmental stages of the potato suggests that these genes are stage-specific in their expression, and we tentatively concluded that this stage-specificity enables the different tissues and organs of the plant to occur better. To analyze the expression differences in *StPIN4*, different tissues were collected for a tissue-specific expression analysis, and the results showed that the target gene was most highly expressed in roots (Figure 7), followed by stems and leaves, and least in tubers. The *PIN* family genes showed significant tissue differential expression, suggesting that these genes play an important role in potato root development and local accumulation of growth factors.

Growth hormones play an important coordinating role in balancing primary and lateral root genesis in plants [35]. Specifically, *PIN1*, *PIN2*, *PIN3*, *PIN4*, and *PIN7*, determine the direction and extent of cell division in root meristematic tissues and the formation of root patterns [36,37,38,39,40,41,42]. It has been shown that in *Arabidopsis*, *AtPIN7* negatively controls root growth and is inhibited to some extent with increasing growth hormone concentration [43]. *HAT2* inhibits root growth through osmoregulation, and *HAT2* regulates growth hormone activity through direct inhibition of *PIN3* transcription [44]. In this study, we obtained transgenic lines by constructing the interference expression vector and overexpression vector of *StPIN4* to transform the potato and further characterized the transgenic plants by qRT-PCR (Figure 8). The obtained transgenic plants were planted in 10 cm × 10 cm plastic pots and continued to be cultured for 21 days to observe their phenotypes, which showed that the suppressed transgenic plants exhibited relatively greater variability in root growth (Figure 9), with the interfering plants showing significant growth in the root system and an increase in the number of lateral roots compared to the overexpressing and non-transgenic plants. In contrast, the overexpression lines phenotypically showed that the growth of the root system of the plants was somewhat inhibited due to the increase in the target gene *StPIN4*, and the number of lateral roots of the plants was somewhat reduced compared with the interfering lines (Figure 10). This is consistent with previous findings that over-accumulation of the growth hormone *PIN* in the roots causes changes in plant root growth as well as in the number of lateral roots [45].

## 4. Materials and Methods

### 4.1. Plant Materials

The in vitro plantlets of the potato variety ‘Atlantic’ were kept and provided by the laboratory of the Potato Biotechnology Innovation Team of Gansu Agricultural University. The in vitro plantlets of the potato variety ‘Atlantic’ were used as experimental materials, and the stem segments with axillary buds of about 2 cm were cut and inoculated in 3% MS solid medium, and then placed in a constant temperature incubator at 24 °C to grow under the following conditions: 20,000 lx, 16 h light, and 8 h dark, with a growth cycle of 21 days. All materials were frozen in liquid nitrogen immediately after harvesting for subsequent experiments.

### 4.2. Identification and Annotation of Potato PIN Gene Family Members

The potato genome sequence was obtained from the potato database Spud DB Potato Genomics Resource, and the Arabidopsis PIN protein family sequences were obtained from the Arabidopsis Genome Database (https://www.arabidopsis.org/index.jsp accessed on 4 October 2024). All the downloaded protein sequences were blasted in the potato database, and all the downloaded protein sequences were screened to identify the potato *PIN* genes by constructing a Hidden Markov Model, and using the model of the Pfam database (http://pfam.xfam.org/) containing the Mem_trans domain (PF03547) to construct a comparative library with the whole genome sequence of the potato to identify the potato *PIN* genes, and then 10 members of the potato *PIN* family were identified, and then numbered and named by chromosome position information. We finally identified 10 members of the potato *PIN* family, and then numbered and named them according to their chromosome position information.

### 4.3. Bioinformatics Analysis of StPINs

Physicochemical traits such as number of amino acids (aa), protein molecular mass (kDa) and isoelectric point (pI) of the potato *PIN* gene family were analyzed using ExPASy (https://www.expasy.org/), and subcellular localization was predicted using PSORT (http://psort.hgc.jp/); transmembrane structural analyses were performed using HMMTOP (http://www.enzim.hu/hmmtop/index.php accessed on 8 April 2022) for transmembrane structure analysis. GSDS was used to obtain the number and distribution of introns and exons of the family members, MEME was used for protein motif analysis, and MEGA 7.0 neighbor-joining (NJ) was used to construct PIN protein evolutionary trees for potato, *Arabidopsis thaliana*, rice, and tomato. The rice PIN protein family sequences were obtained from the Rice Genome Database (http://rice.plantbiology.msu.edu/ accessed on 12 July 2024), and the tomato PIN protein family sequences were obtained from the *Solanum lycopersicum* Genome Database (https://solgenomics.net/search/locus accessed on 11 May 2023). The *PIN* genes were characterized by chromosome locus analysis using Tbtools (v2.034) software. The chromosomal localization of the obtained *PIN* genes was performed using Tbtools software. The online prediction tool PlantCARE (http://bioinformatics.psb.ugent.be/webtools/plantcare/html/ accessed on 1 January 2002) was used to predict and analyze the sequences of 2000 bp upstream of the obtained potato *PIN* gene and the major cis-acting elements.

### 4.4. Gene Expression Analysis of StPINs

Potato plantlets of the ‘Atlantic’ variety were taken as experimental materials, and the total RNA was extracted at the early stage of root development (1 week after inoculation of isolated seedlings onto fresh medium, primary adventitious roots elongated and lateral roots did not grow) and at the maturity stage (3 weeks after inoculation, adventitious root lengths were basically stable, lateral roots were developed, adventitious roots were developed, and root conformation was stable), and the cDNA was synthesized by reverse transcription using the instructions of the Root RNA Extraction Kit and the FastKing RT Kit (with gDNase), and the qRT-PCR was performed using the SuperReal PreMix Plus (SYBR Green) (Servicebio, China) kit to detect the expression level of each family member at different periods of root development. The SuperReal PreMix Plus (SYBR Green) kit was used for qRT-PCR to detect the expression levels among family members at different periods of root development. The reaction system and conditions were strictly executed according to the instructions of the kit, and three technical replicates were set up for each qRT-PCR reaction, with the *StEF1α* gene as the internal reference gene, and the expression levels of *StPINs* were calculated by the 2^−∆∆CT^ method [46].

### 4.5. Construction of Expression Vectors for Potato Transgenic Plants

*StPIN4*, the most significantly differentially expressed gene during potato root maturation, was selected for further functional studies. The *StPIN4* gene sequence was amplified, and the amplified fragment was cloned into the pCAMBIA1300-35S-EGFP vector to construct the overexpression vector of *StPIN4*. The CDS sequence of the *StPIN4* gene was amplified using specific primers and the gene was cloned by PCR with primers 5′-CGGTACCCGGGGATCCATGATAACTTTATCTGATTTCTACC-3′ (*Bam*H I site shown in bold) and 5′-TGCTCACCATGTCGACTCAAAGTCCCATAAATATGTAATAC 3′ (*Sal* I sites are shown in bold). The interference vector for *StPIN4* was constructed by artificial miRNA technology according to the previously published method. The potato variety ‘Atlantic‘ was selected as the transformed recipient material, and the genetic transformation was carried out according to the Si method [47], and the candidate transformed plants obtained were further molecularly verified.

### 4.6. RNA Extraction and qRT-PCR

The total RNA from transgenic and wild-type (WT) plants was extracted according to the instructions of Genes and TRNzol Universal Extraction Reagent, and then PCR was carried out to verify the purity and integrity of the extracted RNA: the above extracted RNA was reverse-transcribed to synthesize the first strand of cDNA according to the instructions of the Servicebio Reverse Transcription Kit, and then the cDNA was used as a template. The cDNA was then used as a template, and the concentration of cDNA was diluted to 100 ng/µL. The qRT-PCR was performed on the key genes using Light Cycler 96 SW1.1. (Roche, Switzerland).

### 4.7. Analysis of StPIN4 Regulates Potato Root Development

Plantlets of WT, overexpression plants, and suppression plants were grown in 10 cm × 10 cm plastic pots for about 21 days, watered every 5 days, and then gently dug up and shook off the soil from the roots after 45 days. Three plants from each line were selected and rinsed under running water. Morphological characteristics including the average number of adventitious roots and lateral roots were observed and the average length of roots was calculated. Three biological replications were carried out to calculate the mean values.

### 4.8. Data Statistics

A one-way analysis of variance (ANOVA) was performed, and Student’s *t*-test data were analyzed using Excel2016, SPSS27, and GraphPad Prism8 data analysis software.

## 5. Conclusions

A total of 10 candidate genes were obtained by mining and characterizing members of the potato *PIN* gene family, and a bioinformatics analysis showed that the protein sequences of this family of genes were specifically conserved during evolution. Analysis based on the protein evolution tree of *PINs* showed that *StPINs* were classified into four subfamilies, with similarly conserved structural domains among the members of each group, and that the functions they exerted were certain. The promoters of *StPINs* are rich in *cis*-regulatory elements related to hormones, light, growth and development, and stress responses. Tissue expression analysis showed that *StPIN4* was highly expressed in roots, suggesting that *StPIN4* plays a key role in regulating root morphogenesis in potatoes. Interference and overexpression vectors for *StPIN4* were successfully constructed, and the transformed plants were obtained through *Agrobacterium*-mediated potato transformation. The results showed that compared with the control non-transgenic strain, the root growth of the overexpression lines was obviously inhibited, the root length was obviously shortened, and the number of adventitious lateral roots was obviously reduced. In contrast, the root system of the suppression lines was obviously increased, and the number of lateral roots was also obviously increased. This suggests that *StPIN4* plays an important role in regulating root morphology and conformation development, and it provides a scientific basis for the genetic improvement of the potato.

## Figures and Tables

**Table 1:** Physicochemical property properties of StPINs proteins.

Name	Gene ID	Number of AA	Molecular Weight	pI	Instability Coefficient	Hydrophilicity Index	Subcellular Localization
*StPIN1*	Soltu.DM.01G025960.1	235	25,398.31	9.22	42.42	0.793	Plasma Membrane
*StPIN2*	Soltu.DM.01G025980.1	355	39,264.82	9.13	36.26	0.762	Plasma Membrane
*StPIN3*	Soltu.DM.02G032050.1	358	39,084.99	9.18	34.90	0.793	Plasma Membrane
*StPIN4*	Soltu.DM.03G033110.1	614	67,134.08	9.08	37.51	0.030	Plasma Membrane
*StPIN5*	Soltu.DM.04G002690.1	622	68,279.87	6.57	39.89	0.141	Plasma Membrane
*StPIN6*	Soltu.DM.04G020760.1	349	38,620.75	6.58	35.82	0.681	Plasma Membrane
*StPIN7*	Soltu.DM.05G004900.1	745	81,967.28	7.05	41.95	0.085	Plasma Membrane
*StPIN8*	Soltu.DM.06G016050.1	531	57,957.83	8.85	33.17	0.408	Plasma Membrane
*StPIN9*	Soltu.DM.07G002230.1	631	68,666.48	9.14	42.51	0.166	Plasma Membrane
*StPIN10*	Soltu.DM.10G026500.1	587	63,906.07	8.81	34.67	0.233	Plasma Membrane

## Data Availability

Data are contained within the article and Supplementary Materials.

## Supplementary Materials

The following supporting information can be downloaded at: https://www.mdpi.com/article/10.3390/ijms252111517/s1.
