# Supplementary material for: Identification of Potato StPIN Gene Family and Regulation of Root Development by StPIN4
Source: Int J Mol Sci. 2024 Oct 26;25(21):11517. doi: 10.3390/ijms252111517 (PMC11546081; doi:10.3390/ijms252111517)
Supplement: Supplementary file 1 [file ijms-25-11517-s001.zip › ijms-3266787-supplementary.pdf]

# Supplementary materials

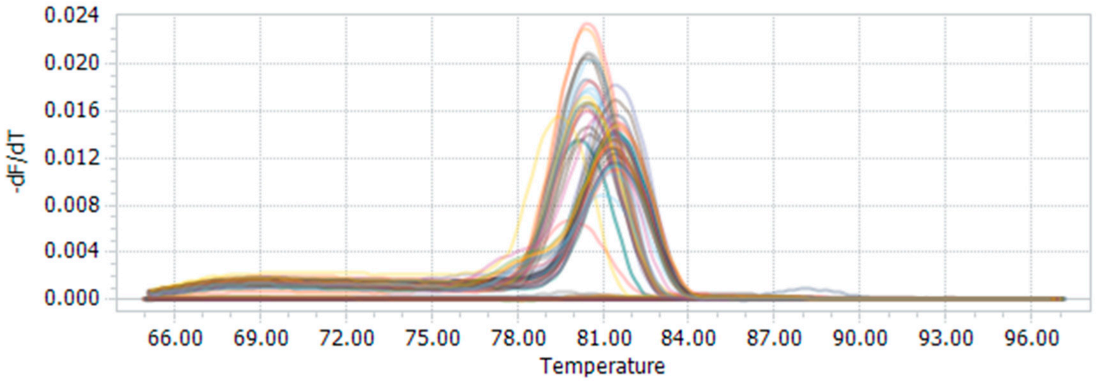


**Figure S1.** qRT-PCR melting curve of transgenic plants

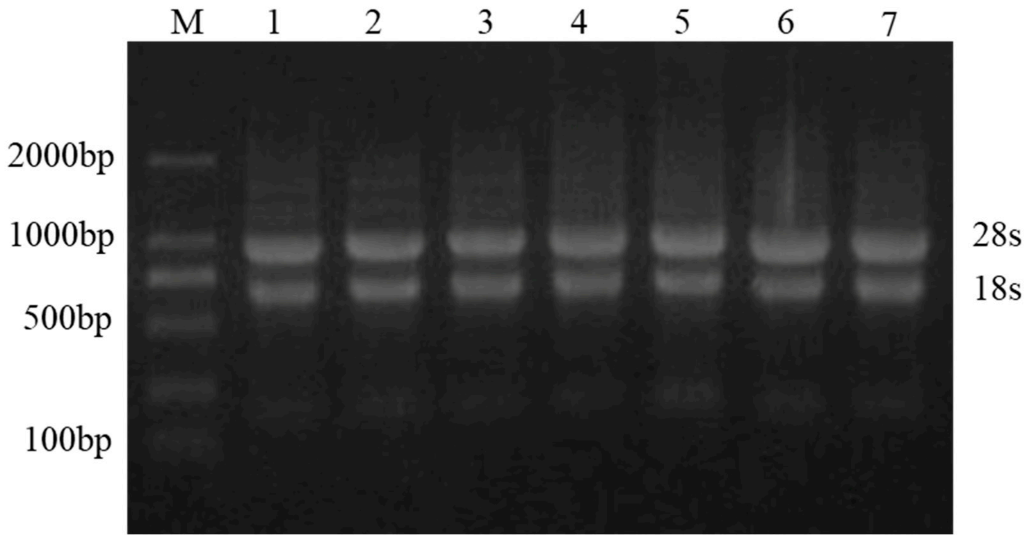


**Figure S2.** RNA PCR detection of transgenic plants

Note：M: DL 2000marker；1-3：Overexpression strains；4：Wild-type lines; 5-7：Interfering expression lines
